# Baastrup’s disease (kissing spines syndrome): a pictorial review

**DOI:** 10.1007/s13244-014-0376-7

**Published:** 2015-01-13

**Authors:** Dimitrios K. Filippiadis, Argyro Mazioti, S. Argentos, G. Anselmetti, O. Papakonstantinou, N. Kelekis, Alexis Kelekis

**Affiliations:** 1 2nd Department of Radiology, University General Hospital “ATTIKON”, 1 Rimini str, 12462 Athens, Greece; 2 GVM Care and Research Maria Pia Hospital, Strada Comunale di Mongreno 180, 10132 Turin, Italy

**Keywords:** Baastrup’s disease, Interspinous bursitis, Imaging, Pain, Spine

## Abstract

**Abstract:**

Excessive lordosis is a common finding and may produce mechanical pressure that causes repetitive strains of the interspinous ligament with subsequent degeneration and collapse. Baastrup’s disease (kissing spine syndrome) is a term referring to close approximation of adjacent spinous processes due to degenerative changes of the spine. Baastrup’s disease usually affects the lumbar spine, with L4-L5 being the most commonly affected level. There is higher occurrence at ages over 70 and no gender predilection. Symptoms include back pain with midline distribution that worsens during extension, is relieved during flexion and is exaggerated upon finger pressure at the level of interest. Diagnosis rests on clinical examination and imaging studies. The hallmark of imaging findings is the close approximation and contact of adjacent spinous processes, with all the subsequent findings including oedema, cystic lesions, sclerosis, flattening and enlargement of the articulating surfaces, bursitis and occasionally epidural cysts or midline epidural fibrotic masses. Proposed therapies include conservative treatment, percutaneous infiltrations or surgical therapies such as excision of the bursa or osteotomy. The purpose of this study is to illustrate the spectrum of imaging findings in Baastrup’s disease and to emphasise upon including the syndrome in the list of potential causes of low-back pain.

**Teaching Points:**

• *Baastrup*’*s disease refers to close approximation of adjacent spinous processes*.

• *Diagnosis of Baastrup*’*s disease is verified with clinical examination and imaging studies*.

• *Contact of adjacent spinous processes results in oedema*, *sclerosis*, *flattening and enlargement*.

• *Proposed therapies include conservative treatment*, *percutaneous infiltrations or surgical therapies*.

## Introduction

Pain due to spinal pathology has a worldwide lifetime prevalence of 54–80 % and an annual prevalence of 15–45 %, with intervertebral discs and facet joints accounting for 26–39 % and 27–40 % of cases respectively [1–3]. However, degenerative disease of the spine is a complex pathology involving not only the vertebral bodies, the intervertebral discs and the facet joints but all the spinal elements (flaval ligaments, interspinous ligaments and posterior vertebral elements) as well. In addition, degeneration of a specific spinal element or a group of elements can result in further degeneration elsewhere in the spine [4]. Correct diagnosis and treatment of spinal pain requires a combination of clinical examination and imaging studies [5]. This combination will reveal the pain source in order for the proper treatment to be selected.

The close approximation of adjacent spinous processes with resultant further degeneration and inflammation was named by Baastrup in 1933 [6–8]. Ever since, these changes are either considered a pathologic syndrome with clinical significance (formation of nociceptors at the level of interspinous contact) or a part of expected degenerative changes occurring with increasing age [8–15].

Baastrup’s disease (also known as “kissing spine syndrome”) is often misdiagnosed, resulting in incorrect treatment and persistence of symptoms. The purpose of this study is to illustrate the spectrum of imaging findings in Baastrup disease and to emphasise upon including the syndrome in the list of potential causes of low-back pain.

## Pathogenesis

Baastrup’s disease is a term referring to close approximation of adjacent spinous processes due to general degenerative changes of the spine. Usually there is an excessive lordosis with resultant mechanical pressure process that causes repetitive strains of the interspinous ligament with subsequent degeneration and collapse [8, 11, 12]. Thus, adjacent spinous processes come in contact and during repetitive shearing movements there is further inflammation of an adventitious bursa present in the interspinous space [8, 11, 12]. Furthermore, these repetitive shearing movements of the closely approximated spinous processes result in additional architectural distortion, flattening, sclerosis and cyst formation in the opposing surfaces [8, 11, 12]. Changes in Baastrup’s disease in most cases occur in association with other degenerative factors such as loss of disc height, spondylolisthesis and spondylosis with osteophyte formation [8, 13]. However, in the literature there are reports in the literature of patients independently developing Baastrup’s disease in the absence of the aforementioned factors [8, 9, 14].

Baastrup’s disease usually affects the lumbar spine with L4-L5 being the most commonly affected level [8, 11]. In most of the cases, only one level is affected and further degenerative changes (such as facet joints hypertrophy, intervertebral disc herniation or spondylolisthesis) can be seen in this pathological level [8]. Concerning the frequency of Baastrup’s disease, studies show a decade-on-decade increase with higher occurrence at ages over 70 and no gender predilection [8, 11].

## Clinical findings

Patients report midline pain which radiates cephalad and caudal but not lateral and medial. Pain due to Baastrup’s disease is aggravated during extension and relieved during flexion [12]. During clinical examination pain is reproduced upon finger pressure at the level of pathologic interspinous ligament. Rarely, in addition to pain there is neurogenic claudication as well, which is associated to the extension of the disease inside the spinal canal (e.g. as an epidural cystic mass) [15, 16]. Upon suspicion of diagnosis, verification is associated to findings of imaging studies (X-rays of the spine, computed tomography [CT], magnetic resonance imaging [MRI] and positron emission tomography [PET-CT]). These imaging studies can be either solely performed or combined in order to illustrate in details the degenerative and inflammatory signs at the level of interspinous ligament.

## Imaging findings

### X-rays

**Fig. 1:**
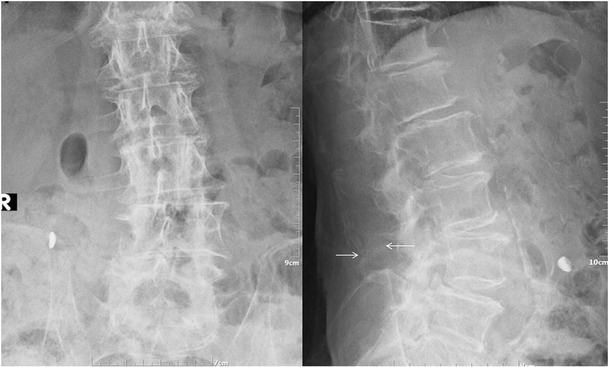
Lumbar spine X-ray, AP (*left image*) and lateral (*right image*) views illustrating close approximation and contact of spinous processes at L4-L5 level with sclerosis and flattening of the articulating surfaces (white arrow)

In standard lateral X-rays of the spine the most common finding is the close approximation and contact of adjacent spinous processes with sclerosis of the articulating surfaces [17, 18]. Additionally, in more severe cases there is flattening and enlargement of the articulating surfaces or articulation of the two affected spinous processes (Fig. 1). Furthermore, general degenerative changes in the spine can be seen, usually most prominent at the pathological level. Advantages of radiographic imaging include the low cost and wide availability of the technique and a relatively low ionising radiation dose. On the other hand, there is poor imaging quality especially at the lower lumbar levels of obese patients.

### CT

**Fig. 2:**
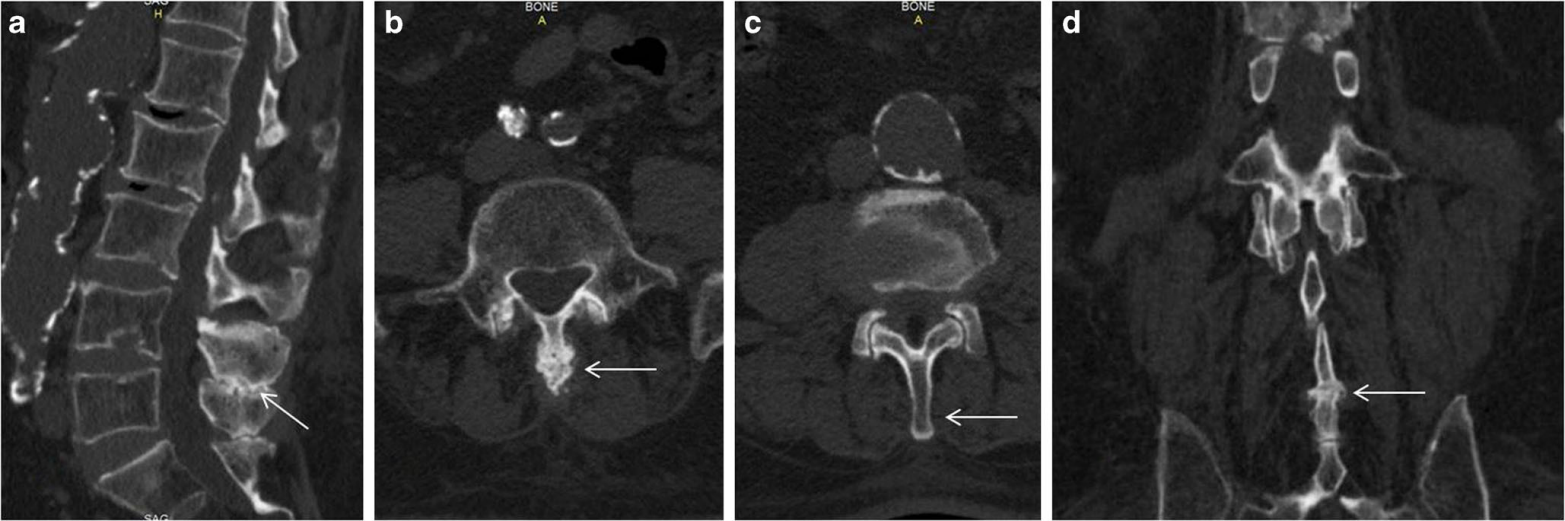
CT images. **a** Sagittal reconstruction of the lumbar spine illustrating close approximation and contact of spinous processes at L4-L5 level with sclerosis, flattening and enlargement of the articulating surfaces (*white arrow*). **b** Axial image in the same patient illustrating nodular enlargement of the L4 spinous process (*white arrow*). **c** Axial image in the same patient illustrating normal architecture of the L2 spinous process (*white arrow*). **d** Coronal reconstruction in the same patient illustrating nodular enlargement of the L4 spinous process (*white arrow*)

**Fig. 3:**
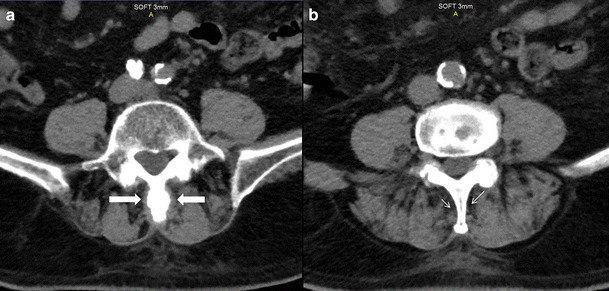
CT images (same patient with as in Fig. 2). **a** Axial image (soft tissue window) illustrating soft tissue nodules (*thick white arrows*) on the sides of L4 spinous process which could represent dissection of the spinal bursa superficial to the erector spinae. **b** Axial image (soft tissue window) in the same patient illustrating normal fat planes (*thin white arrows*) along the L2 spinous process

Axial images with sagittal and coronal reconstructions illustrate in details the close approximation and contact of adjacent spinous processes with additional sclerosis, flattening and enlargement of the articulating surfaces or the articulation of the two affected spinous processes (Fig. 2). Rarely soft tissue nodules are illustrated on the sides of spinous process which could represent dissection of the spinal bursa superficial to the erector spinae (Fig. 3) [19]. CT is able to show degenerative changes (e.g. facet joints hypertrophy, intervertebral disc herniation or spondylolisthesis) in more detail. CT is more expensive than radiographic imaging and is governed by higher ionising radiation dose however, there is also wide availability and in addition, excellent imaging details illustrated in axial, coronal and sagittal levels mainly of the osseous elements. Disadvantages of CT include the poor imaging of interspinous bursae and the limited assessment of intervertebral disc degeneration [5].

### MRI

**Fig. 4:**
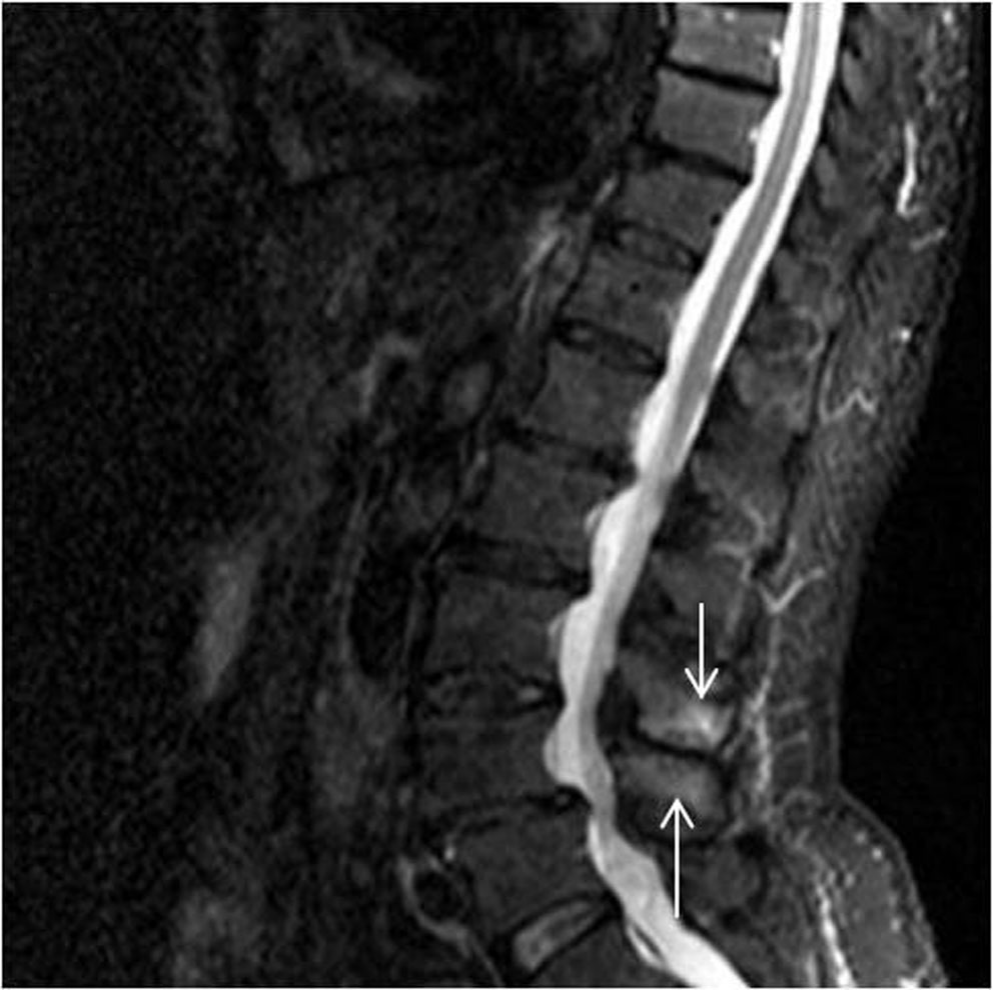
MRI, STIR sequence, sagittal reconstruction illustrating bone oedema at both the spinous processes of L3-L4 level (*white arrow*)

**Fig. 5:**
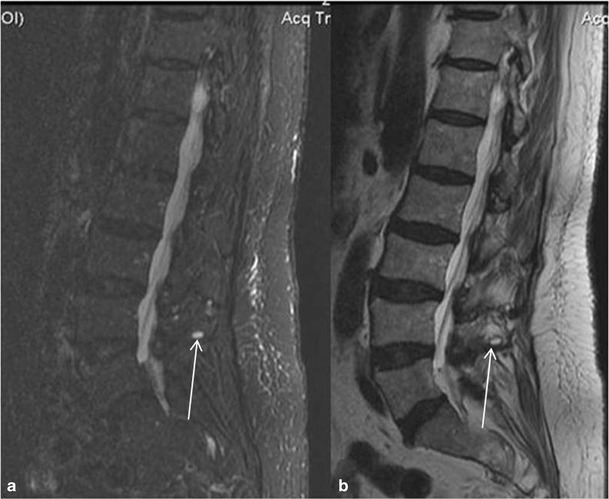
MRI, sagittal reconstruction. **a** STIR sequence illustrating cystic lesion at the articulating surface of L4 spinous process (*white arrow*). **b** T2-weighted sequence (same patient) illustrating cystic lesion at the articulating surface of L4 spinous process (*white arrow*)

**Fig. 6:**
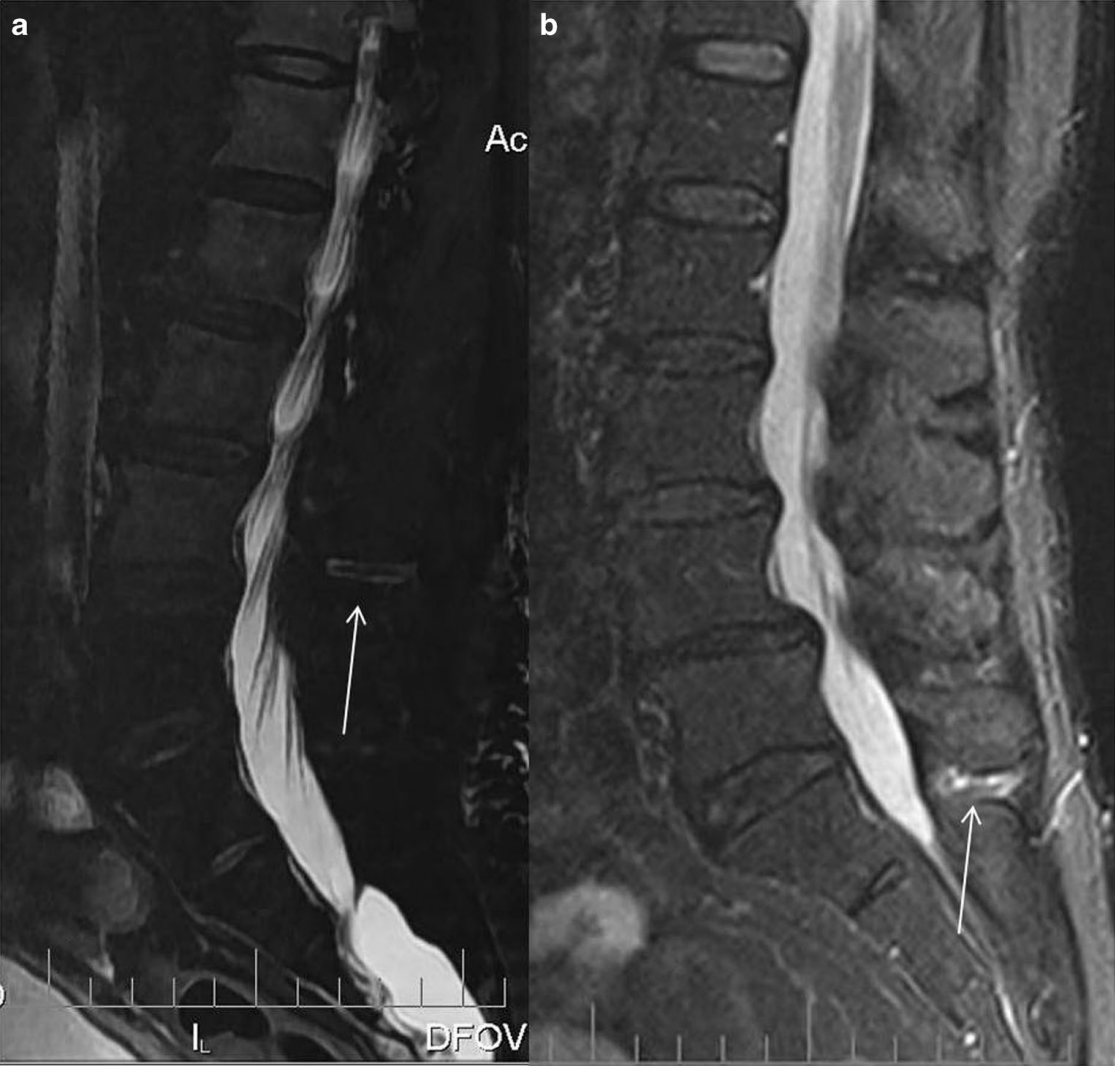
MRI: STIR sequence, sagittal reconstruction illustrating high signal intensity at the interspinous ligament of L3-L4 (**a**) and L5-SI (**b**) level (*white arrow*)

**Fig. 7:**
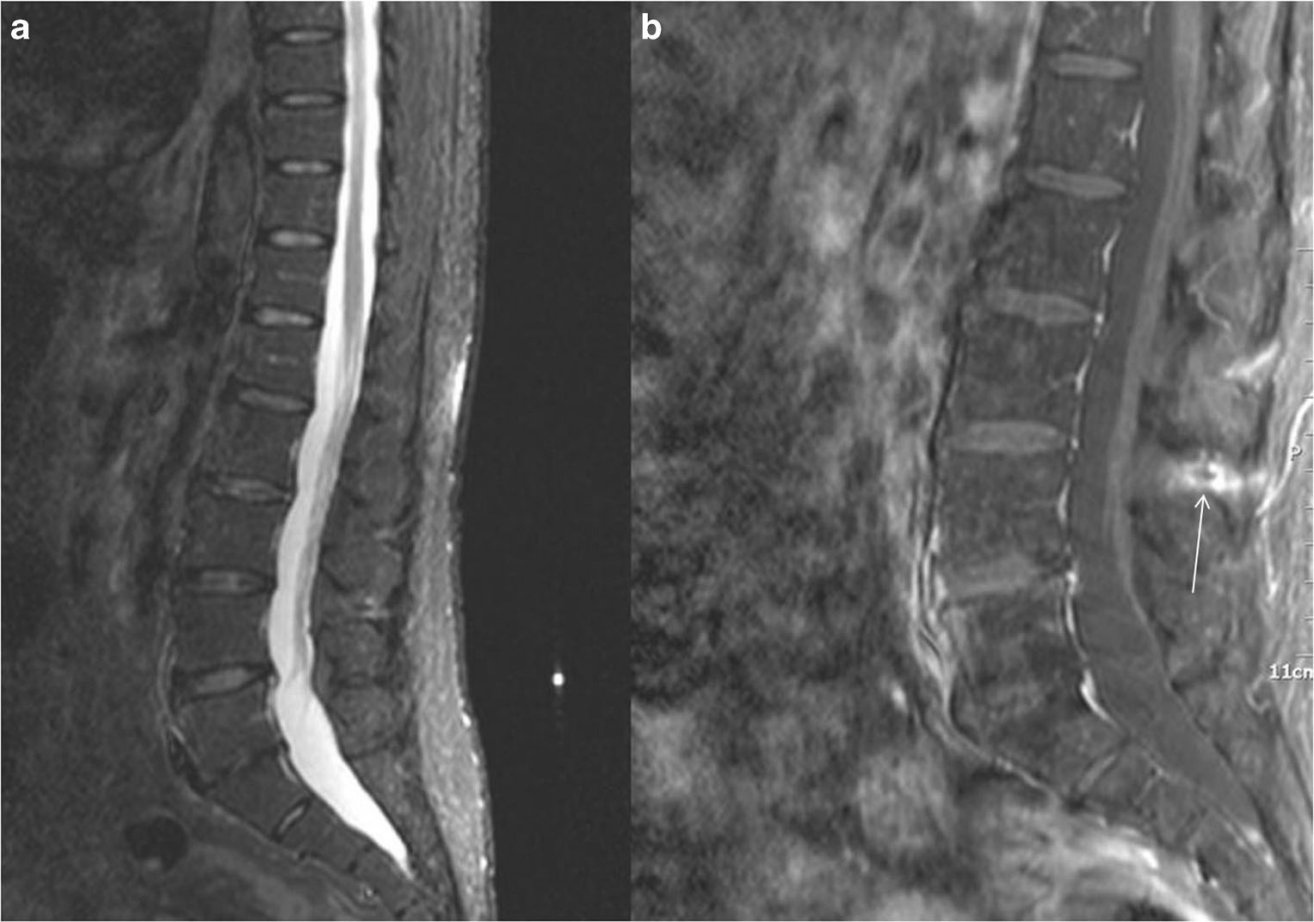
MRI, sagittal reconstruction: STIR sequence (**a**) illustrating high signal intensity and fat-suppression T1-weighted sequence, post intravenous gadolinium injection (**b**) illustrating uptake of the contrast medium at the L3-L4 interspinous ligament (*white arrow*)

In <10 % of the patients with symptomatic Baastrup’s disease MR images reveal lumbar interspinous bursitis, which is illustrated as a fluid-like signal located between the pathological adjacent spinous processes [11]. Additional imaging findings include flattening, sclerosis, enlargement, cystic lesions and bone oedema and at the articulating surfaces of the two affected spinous processes (Figs. 4 and 5). Furthermore, between the pathologic adjacent spinous processes MRI can illustrate oedema at the level of interspinous ligament and enhancement post intravenous gadolinium administration (Figs. 6 and 7). There are reports in the literature of Baastrup disease associated to epidural cysts that cause dural compression or to midline epidural fibrotic mass [15, 16]. Advantages of MRI scan include the lack of ionising radiation and the excellent detailed imaging in axial, coronal and sagittal levels [5]. In addition, there is detailed imaging of the rest degenerative findings and their potential effect upon the spinal cord or nerve roots [5].

## Treatment

Proposed therapies include conservative course of analgesics and non-steroid anti-inflammatory drugs, percutaneous infiltrations with long acting corticosteroids mixed to local anaesthetic or surgical therapies such as excision of the bursa or osteotomy [8, 9, 13, 15, 16, 19]. Specifically for the percutaneous infiltrations, imaging guidance ensures accurate needle positioning with resultant increase of technical and clinical efficacy and at the same time decrease of potential complications rate [20]. Surgery with either partial or total excision of the spinous processes does not always result in pain alleviation [13]. The moderate efficacy of surgical approaches led certain investigators to the theory that kissing spine syndrome is not an actual disease but rather a part of degenerative spinal changes, mainly spondylosis with osteophyte formation [10, 13]. More recent studies suggest that Baastrup’s sign should raise the suspicion of a bursitis [11].

In certain degenerative disorders of the spine, the initial pain reductive effect of conservative therapy does not last and there are relapses in long term follow-up since the causative factor(s) has not been treated [21]. Diagnosis of Baastrup’s disease is important in case of minimally invasive imaging-guided or surgical therapies. In case of misdiagnosis (e.g. facet joint syndrome or intervertebral disc pathology) one will respectively perform intra-articular or epidural infiltration rather than injection at the level of interspinous ligament.

## Conclusions

Baastrup’s disease (Baastrup’s sign, kissing spine syndrome) should be included in the list of potential causes of low-back pain. Imaging findings can be illustrated with various techniques (X-rays of the spine, CT, MRI, scintigraphy and PET-CT), which can be performed either solely or in combination. The hallmark of imaging findings is the close approximation and contact of adjacent spinous processes with all the subsequent findings, including oedema, cystic lesions, sclerosis, flattening and enlargement of the articulating surfaces, bursitis and occasionally epidural cysts or midline epidural fibrotic masses.
